# Malaria Policy Advisory Committee to the WHO: conclusions and recommendations of September 2013 meeting

**DOI:** 10.1186/1475-2875-12-456

**Published:** 2013-12-20

**Affiliations:** 1 Global Malaria Programme, World Health Organization, 20 Avenue Appia 27, Geneva, CH-1211, Switzerland

**Keywords:** WHO, Malaria, Policy making, Mosquito control, Pregnancy, Prevention, Sulphadoxine-pyrimethamine, Treatment efficacy, Drug resistance, Surveillance, Elimination, Plasmodium falciparum, Plasmodium vivax

## Abstract

The Malaria Policy Advisory Committee to the World Health Organization held its fourth meeting in Geneva, Switzerland from 11 to 13 September, 2013. This article provides a summary of the discussions, conclusions and recommendations from that meeting.

Meeting sessions included: recommendations for achieving universal coverage of long-lasting insecticide-treated nets; guidance on estimating the longevity of insecticide-treated nets; improving capacity in entomology and vector control; a review of the latest evidence on intermittent preventive treatment in pregnancy; improving dissemination of Malaria Policy Advisory Committee guidance; updates on the development of the global technical strategy for malaria control and elimination (2016–2025) and the global strategy for control and elimination of *Plasmodium vivax*; updates from the drug resistance and containment technical expert group, the evidence review group on malaria burden estimation, a consultation on malaria case management indicators, and the constitution of the surveillance, monitoring and evaluation technical expert group; subnational elimination criteria; and consideration for future evidence review groups, including diagnosis in low transmission settings and testing for Glucose-6-Phosphate Dehydrogenase Deficiency.

Policy statements, position statements and guidelines that arise from the Malaria Policy Advisory Committee meeting conclusions and recommendations will be formally issued and disseminated to World Health Organization Member States by the World Health Organization Global Malaria Programme.

## Background

The Malaria Policy Advisory Committee (MPAC) to the WHO held its fourth meeting from 11 to 13 September 2013 in Geneva, Switzerland, following its meetings in February and September 2012, and March 2013 [1-3]. This article provides a summary of the discussions, conclusions and recommendations from that meeting^a^ as part of the *Malaria Journal* thematic series “WHO global malaria recommendations” [4].

The following sections of this article provide details and references for the background documents presented at the open meeting sessions of the committee on: recommendations for achieving universal coverage of long-lasting insecticide-treated nets; guidance on estimating the longevity of insecticide-treated nets; improving capacity in entomology and vector control; a review of the latest evidence on intermittent preventive treatment in pregnancy; updates on the development of the global technical strategy for malaria control and elimination (2016–2025) and the global strategy for the control and elimination of *Plasmodium vivax*; updates from the drug resistance and containment technical expert group, the evidence review group on malaria burden estimation, a consultation on malaria case management indicators, and the constitution of the surveillance, monitoring and evaluation technical expert group; subnational elimination criteria; and consideration for future evidence review groups, including diagnosis in low transmission settings and G6PD testing.

The MPAC discussion and recommendations related to these topics, which took place partially in closed session, are also included. MPAC decisions are reached by consensus [5]. The next meeting of the MPAC will be 12 to 14 March, 2014 [6].

### Report from the WHO global malaria programme

The Director of the WHO Global Malaria Programme (WHO-GMP) updated MPAC members on the publications and major activities of each of the WHO-GMP units: vector control; diagnosis, treatment and vaccines; drug resistance and control; and strategy, economics and evaluation [7]. Topics that were later agenda items during the MPAC meeting were not expanded upon during the presentation; the following summary contains highlights of the latest news from WHO-GMP.

Recent vector control documents published by WHO-GMP for use by national malaria control programmes (NMCPs) and partners include: (a) test procedures for insecticide resistance monitoring [8], which are critical for implementation of actions called for in the Global Plan for Insecticide Resistance Management (GPIRM) [9]; (b) an operational manual on indoor residual spraying (IRS) [10]; (c) an operational manual on larval source management [11] as a supplementary measure for malaria vector control in those areas where it is appropriate; and, (d) a handbook for malaria control in humanitarian emergencies [12].

With regard to diagnostic testing, Round 5 of the WHO product testing of rapid diagnostic tests (RDT) [13] is proceeding well; data collection is expected to end in November 2013, with publication of the report in April 2014. In addition, field studies of positive control wells developed by the Foundation for Innovative New Diagnostics (FIND) and Reametrix are currently underway in Uganda and Laos to evaluate their use, utility and acceptability for the quality control of RDTs in routine health care settings in malaria-endemic areas; data collection will be completed by the end of 2013.

Regarding progress with updating the WHO Guidelines for the Treatment of Malaria [14], the systematic reviews for updated guidance are proceeding on schedule and the Chemotherapy Technical Expert Group (TEG) is meeting in November 2013 to review results. A near final draft of the guidelines will be presented to MPAC at its next meeting in March 2014 prior to undergoing internal publication clearance processes within WHO; publication and dissemination is expected to take place in mid 2014.

The WHO-GMP Director also updated MPAC on the latest developments for Seasonal Malaria Chemoprevention (SMC) [15]. SMC was recommended by MPAC at its inaugural meeting in 2012. Since then, three workshops have been organized by WHO in collaboration with the Université Cheikh Anta Diop in Senegal, the London School of Hygiene and Tropical Medicine, and the Roll Back Malaria (RBM) West African Regional Network. These meetings have provided countries with support and helped guide SMC planning and implementation. Nine countries out of 14, where the intervention is potentially appropriate using amodiaquine plus sulphadoxine-pyrimethamine, have adopted and added SMC to their malaria control strategies. Based on their implementation plans, 19 million children could potentially benefit from SMC during the next three malaria seasons, ie., 2013–2015. Unfortunately, large-scale implementation in these nine countries has yet to start due to funding constraints, although small-scale implementation has begun in four of them - Mali, Senegal, Niger, and Nigeria. A field guide to SMC implementation was published in French in August 2013 and both it and the previously released English version are now available on the WHO-GMP website [16].

With regard to training, WHO conducted five courses between June and September 2013, primarily in Africa and the Commonwealth of Independent States (CIS), covering nearly 100 national malaria programme staff, on topics ranging from surveillance, monitoring and evaluation to the prevention of re-introduction of malaria. In August 2013, WHO-GMP published malaria training modules on case management [17] and entomology and vector control [18]; they are available for download from the document centre of the WHO-GMP website.

On drug resistance and containment, WHO, together with affected countries, developed the Emergency Response to Artemisinin Resistance (ERAR) in the Greater Mekong subregion [19]. This regional framework for action from 2013 to 2015 is in line with Global Plan for Artemisinin Resistance Containment (GPARC) [20] recommendations. It was launched on World Malaria Day in April 2013 in Phnom Penh, Cambodia, where WHO has now opened a new regional hub to coordinate response efforts. The aim of ERAR is not to replace existing national, regional or global strategies, but to increase the coordination, quality and coverage of interventions in the Greater Mekong subregion. MPAC welcomed this coordinated approach, and praised the Global Fund for its commitment of $100 million to containing artemisinin resistance in the subregion. However, they expressed concern at the news of continued production of oral artemisinin-based monotherapy, the use of which increases the risk of the spread of artemisinin resistance. They urged the National Drug Regulatory Authorities of the 13 countries (Angola, Bolivia, Cape Verde, Colombia, Equatorial Guinea, Gambia, Myanmar, Papua New Guinea, Sao Tome and Principe, Somalia, Swaziland, Timor Leste, and Vanuatu) that still allow the marketing of oral artemisinin-based monotherapy medicines to ban their sale in order to help lower demand for continued production.

A core role of WHO-GMP is to keep an independent score of global progress in malaria control and elimination [21]. One of the ways it does this is via the annual World Malaria Report (WMR) [22], which will be launched this year on 11 December in Washington, D.C. WHO-GMP is also finalizing an updated Malaria Programme Review manual after extensive input from partners, which will be a much simpler version of the edition released in 2010 [23]. This development was strongly welcomed by MPAC, who noted that NMCPs need a simple and useful way of measuring and reviewing their control and elimination programme performance. They also welcomed progress with the Malaria Situation Room [24], which was formally launched at the Special Summit of the African Union on HIV/AIDS, Tuberculosis and Malaria in Abuja, Nigeria in July 2013; it is a joint initiative of WHO, RBM, the African Leaders Malaria Alliance, the Office of the UN Secretary-General’s Special Envoy for Financing the Health Millennium Development Goals and for Malaria, as well as the International Federation of Red Cross and Red Crescent Societies. The Malaria Situation Room identifies bottlenecks to achieving universal access to malaria control and finds solutions in the ten countries with the highest malaria burden in Africa: Nigeria, the Democratic Republic of the Congo, the United Republic of Tanzania, Uganda, Mozambique, Côte d’Ivoire, Ghana, Burkina Faso, Cameroon and Niger. Together these countries account for more than 70% of Africa’s malaria burden, and 56% of the global malaria burden. The Global Fund, the US President’s Malaria Initiative and UNICEF have recently joined the Situation Room and are contributing to the weekly calls.

MPAC commended the work of WHO-GMP and their partners in the global malaria community in supporting countries in their efforts to monitor and reduce their malaria burden. The next report from WHO-GMP to MPAC in March 2014 will focus on the key findings from the World Malaria Report 2013.

### Universal coverage of long-lasting insecticide-treated nets

Following its establishment at the September 2012 MPAC meeting, and the subsequent open call and selection process for members in early 2013 [25], the Vector Control TEG (VC TEG) met for the first time in July 2013 to begin its task of reviewing and making recommendations on the use and appropriate mix of vector control interventions in the control and elimination of malaria [26]. A major output from that meeting was a report to MPAC with recommendations on methods for achieving and sustaining universal coverage of long-lasting insecticidal nets (LLIN) [27,28].

LLINs have played an important role in the remarkable success in reducing malaria burden over the past decade [22]. They are a core prevention tool and are widely used by people at risk of malaria. However, LLINs wear out gradually over time and need to be replaced. Therefore, sustaining universal LLIN coverage remains challenging for many countries. The VC TEG considered how universal coverage, defined as universal access to and use of LLINs, can be achieved and sustained operationally.

Among the conclusions of the VC TEG was that in order to maintain universal coverage, countries should apply a combination of mass distribution and continuous distribution through multiple channels, in particular antenatal and immunization services. The term “continuous” was defined as distribution systems that deliver nets continuously and without interruption over time, as opposed to “campaigns” which deliver a consignment of nets to a defined target population in a single time-limited operation. The VC TEG recommended that mass campaigns should be repeated, normally at an interval of no more than three years, unless there is reliable evidence that a different interval would be appropriate. They also recommended that continuous distribution channels should be functional before, during and after the mass distribution campaigns to avoid any gap in universal access to LLINs.

The VC TEG recommended that there should be a single national plan, under the leadership of the NMCP, for both continuous and campaign distribution strategies. This unified plan should include a comprehensive quantification and gap analysis for all public sector LLIN distribution channels. In addition, each NMCP should develop its own LLIN distribution strategy, based on an analysis of local opportunities and constraints. The strategy should identify a combination of cost-effective and equitable distribution channels to achieve and sustain universal coverage, which in addition to mass campaigns and continuous distribution through antenatal clinics (ANC) and the Expanded Programme on Immunization (EPI), could include channels such as schools, community-based platforms, religious networks, agricultural and food-security support schemes, and the private and commercial sector. Ministries of Health should ensure that NMCPs have adequate human and financial resources for efficient programme management, as well as for LLIN procurement and distribution.

The VC TEG also recommended that because the lifespan of LLINs vary widely among different nets and settings, making it difficult to plan the frequency at which replacement nets need to be procured and delivered, all LLIN programmes should carry out durability monitoring using WHO guidance [29]. In addition, there should be efforts to improve LLINs through repair of small holes before they become bigger, as well as behaviour change interventions to improve net longevity and use.

It is important to note that the VC TEG did not recommend periodic “top-up campaigns”. However, the VC TEG suggested that a NMCP could consider top-up strategies (rather than full replacement without taking current net ownership into account) if 40% or more of the target population have LLINs that are less than two years old.

MPAC fully endorsed the VC TEG’s recommendations for sustaining universal coverage of LLINs, as well as the indicators suggested for monitoring progress towards universal coverage (e g, repeated longitudinal estimates of the percentage of the population with access to a LLIN within the household). The report was approved pending edits to improve the clarity and conciseness of the document prior to WHO publication. These edits were adopted and the WHO recommendations for achieving and sustaining universal coverage of LLINs are now available on the WHO-GMP website [30].

### Estimating the longevity of insecticide-treated nets

The VC TEG presented MPAC with a report and draft guidance note on estimating the longevity of LLINs for malaria control [31,32]. The durability of LLINs in the field has become a critical issue for the success of malaria control in areas where LLINs are being applied for malaria prevention for two main reasons: (a) it has been shown by various modelling exercises that increasing LLIN durability by one or two years on average would have a huge impact on the cost of malaria prevention, in the order of $500-700 million savings over five years; and, (b) there are increasing data suggesting that there is a wide variation of LLIN durability between different locations or populations. This implies a need to acquire country- or region-specific data on LLIN performance so that LLIN procurement decisions can be based on price per year of protection rather than unit price per net.

The VC TEG noted that significant progress has been made towards performance-based procurement with the release in 2011 of the “Guidelines for monitoring the durability of LLINs under operational conditions” by WHO [29], which not only addresses some of the methodological issues but also encourages countries to incorporate assessment of LLIN performance as part of their distribution efforts. Furthermore, the concept note on a system to improve LLIN procurement through market competition [33] issued by WHO in 2011 clearly states the importance of “value for money” and that “for LLINs, the criteria for comparison could be 'cost per median year of net life under local conditions of use’”. However, the current guidelines are not comprehensive enough to allow countries that have already started to collect data on LLIN performance to translate their findings into the required “median LLIN survival”, and an expansion of these guidelines was, therefore, needed.

LLIN durability and survival depend on two factors: (a) net attrition, ie., complete loss of nets; and, (b) physical integrity, ie., holes and tears in nets still existing in households. Net attrition from households includes both LLINs that are potentially still in use elsewhere (given away for others to use, stolen) and nets that are no longer usable or available (discarded, destroyed, used for other purposes). The expanded guidance from the VC TEG provides country programmes and partners with a method for calculating the functional survival of LLINs from field data obtained from prospective or retrospective surveys, as well as a method to estimate the median survival time of LLINs. These methods are based on the best available evidence to date.

MPAC fully endorsed the VC TEG’s recommendations for estimating the longevity of LLINs, concluding that it will help provide guidance to countries to track LLIN durability in the field in order to support management of resupply and to inform global level procurement decisions in conjunction with urgently needed new, more predictive textile laboratory testing currently under consideration by WHO. Following edits to improve the clarity and conciseness of the guidance note, the WHO guidance on estimating the longevity of LLINs was finalized and is now available on the WHO-GMP website [34].

### Improving capacity in entomology and vector control

The VC TEG presented recommendations for countries and partners to improve capacity in entomology and vector control [35,36]. The VC TEG explained that malaria control is at a critical juncture and that the goal of malaria elimination in many settings might not be achieved without adapting to the changing threats and opportunities for controlling transmission. Progress in global malaria control over the past decade has been largely gained through investments in vector control, especially insecticide-treated mosquito nets (ITNs) and IRS. In order to sustain and build further on these gains, there is a need to improve the efficiency of malaria vector control, including better targeting of interventions, and more effective management of anopheline resistance to insecticides. These challenges can only be met by national staff with the training, support and career structures that allow them to effectively plan, monitor, evaluate, and manage vector control efforts.

The VC TEG recommended that Ministries of Health should ensure that each NMCP has the human capacity and infrastructure to support vector control and entomological monitoring, including monitoring for insecticide resistance. The NMCP should establish or strengthen an intersectoral coordination mechanism to develop a long-range strategic plan for building human resources and systems for public health entomology and vector control. The plan should include: conducting training needs assessments and curricula review for pre-service and in-service training (including epidemiology and management). This will ensure training is directly relevant to the expected skills of staff tasked with entomological monitoring and vector control. The plan should also review, revise or establish posts and career development structures for entomology and vector control specialists at national and subnational levels within Ministries of Health or other appropriate government structures. In cases where basic entomological capacity is lacking within the NMCP, the intersectoral coordination mechanism should include the establishment of agreements with national universities and training and research institutions to provide ongoing training and technical support, including reference laboratory services for entomological monitoring and vector control.

These sentiments on the urgent need for human capacity strengthening in the field were strongly supported by MPAC. They endorsed the VC TEG recommendations, and these are now available on the WHO-GMP website as a WHO guidance note for countries and partners to improve capacity for malaria entomology and vector control [37].

### Intermittent preventive treatment in pregnancy

In October 2012, WHO updated the malaria in pregnancy (MIP) policy for intermittent preventive treatment during pregnancy with sulphadoxine-pyrimethamine (IPTp-SP). WHO recommends that women who live in moderate to high malaria transmission areas should receive IPTp-SP as early as possible in the second trimester and at each scheduled ANC visit thereafter, provided that each SP dose is given at least one month apart [38].

Since the updated IPTp policy was released, multiple countries throughout sub-Saharan Africa have reviewed the new policy and plan to update their country policies and start programme implementation. As a further step in the policy making process, the Evidence Review Group (ERG) on IPTp met from 9 to 11 July, 2013 to assess the results of recently completed multicentre clinical trials on mefloquine use for IPTp (IPTp-MQ), and to review the evidence on the effectiveness of IPTp-SP in relation to *Plasmodium falciparum* antifolate resistance and decreasing malaria transmission [39]. In relation to mefloquine, the specific objectives of the ERG meeting were to review evidence of the efficacy, safety and tolerability of 15 mg/kg MQ for IPTp, given as single or split dose, compared to SP in HIV-negative pregnant women, and the benefit of three monthly doses of IPTp-MQ added to daily co-trimoxazole (CTX) prophylaxis in HIV-infected pregnant women.

The MPAC reviewed the ERG recommendations [39], and agreed that MQ at the 15 mg/kg dose regimen should not be recommended for IPTp given its adverse events and poor tolerability. In relation to SP resistance, MPAC recognized that in many areas where parasites with quintuple mutations conferring antifolate resistance have been identified, IPTp with SP still confers benefit in terms of pregnancy outcomes. In a small number of discrete, limited areas in eastern and southern Africa, resistance of *P. falciparum* to SP has reached a level at which IPTp-SP may no longer be effective in preventing low birth weight. These are areas where there are *P. falciparum* parasites carrying six resistance mutations in *dhfr* and *dhps* genes, including the A581G *dhps* mutation. On balance, MPAC concluded that there is currently insufficient data to determine at what level of resistance IPTp-SP should be discontinued in the absence of an established and effective alternative. Similarly, MPAC concluded that there are currently insufficient data to define the level of *P. falciparum* transmission at which IPTp-SP may cease to be cost-effective from a public health point of view. Furthermore, natural fluctuations in malaria incidence from year to year, and the low cost of the intervention as delivered through the Maternal and Child Health system, call for significant caution before discontinuing IPTp-SP. More data are needed and will be reviewed when available. Until that time, MPAC strongly recommended that countries should continue to implement the current WHO policy that women who live in moderate to high malaria transmission areas should receive IPTp-SP as early as possible in the second trimester, and at each scheduled ANC visit thereafter, provided that each SP dose is given at least one month apart [38]. WHO-GMP has developed a policy brief to provide guidance to national health authorities in Africa to support the implementation of IPTp-SP; it is available on the WHO-GMP website [40].

### Global technical strategy for malaria control and elimination (2016–2015)

In response to a request by MPAC in 2012, and an expression of support by WHO Member States at the 2013 World Health Assembly in May, WHO-GMP is coordinating the development of a Global Technical Strategy for Malaria Control and Elimination (GTS) for 2016–2025. As requested, the GTS will articulate the goal and global targets for malaria control and elimination over the next decade. This will be a unifying document that synthesizes current policy recommendations and comprehensive, evidence-based and cost-effective strategies for WHO Member States to use in developing their own strategies. The document will also provide a platform for ensuring that the impressive gains of the last decade are sustained, and that further progress is accelerated along the pathway to elimination.

The Chair of the recently formed GTS Steering Committee provided an update to MPAC on progress since its last meeting in March 2013 [41]. The Steering Committee, composed of 14 leading malaria technical experts, scientists and representatives of endemic countries, who have been tasked with guiding WHO-GMP on the development of the GTS, leading evidence reviews and ensuring that the process is rigorous and inclusive of national and regional inputs, met for the first time from 29 to 30 July, 2013 in Geneva [42]. The Steering Committee discussed its work plan and the GTS development timeline so that its findings will be ready to be presented to WHO Member States for consideration at the 2015 World Health Assembly.

The GTS will be developed through an inclusive process that draws on existing country and regional strategies as well as consultations with WHO Regions, international experts and country programmes. The key contribution to the GTS development process will be the convening of seven regional expert consultations led by the WHO Regional Offices from February to May 2014. Concurrent with the GTS development process, RBM will develop the Global Malaria Action Plan 2 (GMAP 2), the second generation of an RBM consensus document which provides the global framework for coordinated action by all malaria stakeholders supporting acceleration of malaria control and elimination efforts. The GTS will serve as the technical foundation for the GMAP 2 and the two documents will be developed in a synchronous, collaborative process with shared goals and global targets for malaria over the next decade. At the request of MPAC, a mechanism has been put in place to ensure that the GTS Steering Committee and the RBM GMAP 2 Taskforce work together to ensure full complementarity of both documents. Four members of the GTS Steering Committee and the GMAP 2 Taskforce will sit on both boards and the Executive Director of the RBM Partnership Secretariat and the Director of WHO-GMP will be *ex officio* members of both groups. It is envisioned that the GTS and GMAP 2 will be launched as companion documents in late 2015, after consideration by the World Health Assembly for the GTS and RBM Board adoption of the GMAP 2.

MPAC commended the GTS Steering Committee and WHO-GMP on progress to date, and the leadership of WHO-GMP and RBM on the close alignment of the processes for the GTS and GMAP 2. MPAC members were especially supportive of the inclusive process that will involve country and regional input; these will be central to the development of the GTS and critical for its success. To inform the goal and targets of the GTS, a baseline analysis to look at all existing regional and national malaria strategies is currently underway. The GTS Steering Committee will next update MPAC at its March 2014 meeting in Geneva.

### Global strategic plan for *Plasmodium vivax* malaria

WHO-GMP provided MPAC with an update on progress with the Global Strategic Plan for *Plasmodium vivax* malaria [43,44]. The first writing committee meeting took place in Barcelona on May 31, 2013. An outline was drafted for each of the Thematic Review topics with a focus on programmatic relevance, in particular, biology, epidemiology, vector control, diagnosis and treatment, surveillance and elimination, costs and cost-effectiveness, and research priorities. In addition, ten countries (Azerbaijan, Brazil, Cambodia, China, Ethiopia, India, Indonesia, Iran, Nicaragua and Papua New Guinea) with diverse *P. vivax* endemicity have been selected for Country Landscape Briefs. These briefs will include details of their *P. vivax* epidemiology, interpretation of distributional patterns and trends over time, malaria control interventions (policy and practice) as well as gaps and constraints in relation to WHO policy guidance. Work is in progress and a draft of the Global Strategic Plan is expected in early 2014.

The schedule for developing the *P. vivax* plan has been modified so that it is more closely aligned with the timeline for the GTS, and will, therefore, be better and more fully integrated with it in terms of content. The planned *P. vivax* regional consultations will now take place alongside the GTS regional consultations during the first half of 2014. An update will be provided to MPAC at its next meeting in March 2014.

### Improving dissemination of MPAC guidance

WHO-GMP updated MPAC on work it has undertaken to improve knowledge management across the three levels of the organization (headquarters, regional offices and country offices), in particular to improve the dissemination of policy recommendations and MPAC meeting reports, to strengthen its external and internal communications infrastructure, as well as possible suggestions for MPAC-related information management [45,46].

Over the past year, WHO-GMP has worked on strengthening its knowledge management infrastructure while at the same time improving its presence at high-level governmental events and scientific conferences to generate better visibility for its policy recommendations. The biggest change to date is the upgrading of the external communications architecture, which has the central WHO malaria website [47] at its core.

The malaria website has been updated and re-built in six official WHO languages, with the new content architecture allowing easier access to information on all intervention areas, and providing a clear prioritization of content. New site features include a news archive to track all announcements, a media centre for journalists and the advocacy community, and a document centre containing all malaria documents in one place, with improved navigation and search functionality.

The French content has been significantly expanded, bringing about a major improvement in the way material is presented to NMCPs and partners in Francophone countries. In addition, many documents containing outdated guidance have been archived and taken off third-party sites. The long-term vision is to build a global information hub on malaria that pulls together all of WHO’s malaria information, including information from other programmes and regional sites.

WHO-GMP also provided an update about other dissemination channels, such as document launches at high-level inter-governmental events, information about audience profiles and website statistics, and opportunities for further strengthening of dissemination efforts.

MPAC members enthusiastically welcomed ongoing efforts to improve knowledge management, in particular the changes to the WHO-GMP website. They also welcomed WHO-GMP considering the presentation of content from a multilingual perspective and requested that WHO-GMP consider adding a section that brings together Portuguese-language publications in one place.

### Drug resistance and containment

The Drug Resistance and Containment (DRC) TEG updated MPAC on its 27 to 28 June, 2013 meeting in Geneva [48]. Among the agenda items discussed at that meeting was the Emergency Response to Artemisinin Resistance (ERAR) in the Greater Mekong subregion [19], which is a framework that aims to strengthen current efforts and increase cross-border collaboration between Cambodia, Myanmar, Thailand, and Viet Nam on containing resistance and eventually eliminating malaria. MPAC endorsed the DRC TEG’s recommendations on the current ERAR tier designations. Based on recent study results they recommended that the following additional provinces should be designated as Tier I (ie., areas for which there is credible evidence of artemisinin resistance): Bago East and Kayin provinces in Myanmar, and Preah Vihear province in Cambodia. Kayah province in Myanmar is also likely to meet the Tier I designation, but the recommendation is pending the availability of quality control data from therapeutic efficacy studies. The district of Attapeu in Laos, currently designated as Tier II, may also be changed to Tier I after a review of new data.

The DRC TEG also informed MPAC that the Tracking Resistance to Artemisinin Collaboration (TRAC) study has confirmed areas in Southeast Asia where slow clearance phenotype *P. falciparum* have been identified by high day 3 positivity rates during routine therapeutic efficacy studies. They have also identified new areas where increased vigilance is needed. A comparison of the detailed parasite clearance data from the TRAC studies with that of day 3 positivity rates during therapeutic efficacy studies, supplemented by preliminary modeling results, indicates that the current TEG-recommended threshold of ≥10% day 3 positivity rates for defining suspected artemisinin resistance is still appropriate.

MPAC endorsed the DRC TEG recommendation to identify a replacement for the current first-line treatment, atovaquone-proguanil, for uncomplicated *P. falciparum* malaria in Western Cambodia since it is effectively a monotherapy and hence vulnerable to resistance, illustrated by reports of high failure rates in areas where it has been deployed. The DRC TEG concluded that the best alternative treatment option is the fixed combination of pyronaridine-artesunate, but that a study must be conducted urgently to confirm its efficacy in Western Cambodia. The extension of artemisinin-based combination therapy (ACT) regimens dihydroartemisinin-piperaquine or artemether-lumefantrine from three to either five or seven days could be an alternative option in areas where ACT is failing, but this will also require additional efficacy and safety studies.

Other updates provided to MPAC by the DRC TEG included: a review of suspected artemisinin resistance in Suriname and Guyana; recent developments on assessing parasite clearance; an update on molecular markers for artemisinin resistance; an update on *in vitro* artemisinin susceptibility testing; the outcome of recent modelling efforts on multiple first-line treatments; and developments in the study design and implementation of mass drug administration as a tool for eliminating artemisinin-resistant malaria. Further details about each of these topics are included in the DRC TEG meeting report which is available on the MPAC September 2013 meeting background documents page of the WHO-GMP website [49].

The next meeting of the DRC TEG is scheduled to take place in early 2014, and an update will be provided to MPAC at its next meeting in March 2014.

### Malaria burden estimation

The ERG on malaria burden estimation (ERG MBE) met for the last of its three planned meetings from 8 to 9 July, 2013 [50] to: (a) discuss updates on relevant work since their previous meeting in January 2013; (b) achieve consensus on the methods that should be used by WHO and in the World Malaria Report (WMR) to estimate malaria cases and deaths; and, (c) develop research agendas to improve estimates and address bottlenecks that prevent reconciliation of different methodologies and results.

The ERG MBE Chair presented MPAC with its conclusions and recommendations for WHO malaria morbidity estimates [51]. These included: (a) for the 2013 WMR, WHO-GMP should use the same methodology for case estimation as they currently use. However, for 2014 and after, WHO-GMP should use the Malaria Atlas Project (MAP) “cube” case estimates for African countries without strong surveillance systems; (b) WHO-GMP will need to continue to present time series of cases and deaths in each WMR so that journalists and other consumers of the information will not create their own time series by extracting annual estimates from different WMRs (which will be influenced by changes in methodology and data validation); (c) WHO-GMP should discuss with partners the feasibility of collecting prevalence data through household surveys such as the Malaria Indicator Survey (MIS) on all age groups (not just six to 59 months) so that the age pattern of *P. falciparum* parasite rates (*Pf*PR) can be further examined. The sample of older children and adults available at home at the time of a survey may not be an accurate representation of the population as a whole, but the data would still be useful; (d) the assumptions about parasitaemia and different care-seeking behaviours would benefit from further validation. To do so, the analysis examining parasite prevalence stratified by type of care-seeking behaviour should be supplemented with more recent surveys and surveys from outside of Africa, if available (such as from the WHO Eastern Mediterranean Region). If the analysis indicates highly variable results by region, the assumptions used may need to be country- or region-specific; and, (e) WHO-GMP should report on parasite prevalence as one of their key indicators (in addition to cases and deaths). As with cases and deaths, the WMR will show country-reported parasite prevalence values and modelled parasite prevalence (from MAP). WHO-GMP will need to consider the factors that complicate reported parasitaemia. Since prevalence changes by season, presenting a static annual value may be misleading. In some areas outside of Africa, estimates of cases may be of higher quality than MAP prevalence estimates, so WHO-GMP will need to decide whether to convert case data into prevalence values in order to generate estimates of *Pf*PR for the entire globe. WHO-GMP will then need to determine whether country consultations on *Pf*PR will be required, as with cases and deaths.

MPAC endorsed the ERG MBE’s recommendations and conclusions that generating user-friendly and transparent methodologies for estimates of malaria prevalence, cases and deaths might help increase country participation and ownership over the estimates, which in turn, should encourage more investment in data quality.

Regarding WHO malaria mortality estimates [51], the ERG MBE conclusions and recommendations were that: (a) WHO-GMP should use the same methodology for the 2013 WMR malaria mortality estimates as has previously been used. Once further research is conducted, WHO-GMP may want to change the methodology for estimating malaria deaths, but there is no evidence to justify such a change at the present time; (b) WHO-GMP should also use the same assumptions in the 2013 WMR that have previously been used. In the future, some assumptions, such as a fixed case fatality rate (CFR) for estimates outside Africa, should potentially be revisited. The ERG recommended against applying a case fatality rate to estimate the number of malaria deaths for highly endemic countries in Africa; ERG MBE members felt that it would be difficult to identify an appropriate CFR in the light of changing treatment practices. Of note, in Africa WHO uses verbal autopsies as a key source of information on malaria mortality rates in children under five years of age; (c) WHO-GMP and the global malaria community should be clear that the age group “>5 years” should not be interpreted as meaning “adults” as a significant proportion of morbidity and mortality in this broad age group can refer to persons between five and 15 years of age.

MPAC thanked the ERG MBE for its careful and diligent work over the past year, and particularly to all the researchers with their methodologically diverse backgrounds who participated and actively contributed to the discussions on how to improve malaria estimates now and in the future. MPAC concluded that given that the malaria mortality research agenda is just in its beginning stages, additional meetings of a ERG MBE may be necessary in order to evaluate new methodologies in the future. In the meantime, the Surveillance, Monitoring and Evaluation TEG (SME TEG) would take over the functions of the current ERG, and the ERG MBE would be considered closed. WHO-GMP has already accepted the ERG MBE’s suggestions for improvement to the 2013 WMR, which will be released in December 2013.

### Malaria case management indicators

WHO-GMP presented MPAC with the conclusions from an informal consultation on malaria case management indicators that took place in Geneva from 10 to 11 July, 2013 [52,53]. The meeting brought together experts and WHO technical staff to share current knowledge and practices regarding monitoring malaria case management.

Several recent developments in malaria control policies and practices, for example the 2010 WHO recommendation for universal diagnostic testing of all suspected malaria cases and the 2012 launch of the “T3: Test. Treat. Track” Initiative [54], have highlighted the need for improved monitoring of malaria case management practices since current approaches have important limitations, particularly in high-burden countries. For example, national programme data on diagnostic testing and treatment are rarely linked in a way that facilitates tracking of testing and treatment practices. Although national household surveys are increasingly available, the validity of information on diagnostic testing and treatment collected has been questioned. Health facility-based surveys can address some of the limitations of programme and household survey data on malaria diagnostic testing and treatment, as patient testing and treatment information can be reliably linked and recall bias of respondents can be reduced.

The limitations of current case management indicators were acknowledged by MPAC. Ultimately, routine monitoring needs to be improved; however, the need for better information on malaria case management is acute. As an immediate next step, WHO-GMP will draft a protocol and conduct a pilot using Service Availability and Readiness Assessments (SARA) of health facilities to explore whether using SARAs to collect information on malaria testing and treatment is feasible. If so, the results will be made available and the practice will be promoted more widely. MPAC supported the need to be able to employ more focused, facility-level surveys in the short term while improving routine systems for the long term. They suggested that this area of work should be linked to the terms of reference for the new SME TEG.

### Surveillance, monitoring and evaluation

WHO-GMP updated MPAC on progress with constituting the SME TEG [55]. Since its last meeting and at the request of MPAC, the draft terms of reference for the SME TEG were presented to the RBM Monitoring and Evaluation Reference Group (MERG) in New York in May for input. These were incorporated and presented to MPAC for approval [56].

The SME TEG will report to MPAC and provide advice to WHO on surveillance, monitoring and evaluation at the national, regional and global level. This includes: (a) choice of indicators for monitoring the financing, coverage, quality, and impact of malaria control interventions at the national and global level; (b) strategies for obtaining, synthesizing and disseminating information on the indicators globally, including modelled estimates of intervention coverage and disease burden; (c) guidance that WHO provides on (i) surveillance of infections, cases and deaths and the use of these data in decision-making, (ii) establishing systems for monitoring programme financing and coverage, (iii) evaluating the impact of malaria interventions and programmes; (d) evaluating the accuracy and integrity of SME data at the national, regional and global level; (e) approaches for strengthening the capacity of WHO Member States to generate and use key information; and, (f) identifying gaps in evidence and suggesting priority research areas in the field of SME.

WHO-GMP will soon begin a call for CVs of interested experts for the SME TEG and will constitute the group in early 2014. It is scheduled to meet for the first time in the first half of 2014, and an update will be provided to MPAC at its next meeting in March 2014.

### Subnational elimination criteria

Some countries have undertaken malaria elimination at subnational level. For example, in the Philippines, 27 out of 80 provinces have been declared malaria-free to date. However, there are no global guidelines for achieving and validating malaria-free status in smaller subnational geographic areas such as states, regions or provinces. At its last meeting, MPAC concluded that subnational elimination targets, should countries choose to pursue them, could be important internal milestones for countries, as well as being potentially important international milestones, especially in larger countries.

WHO-GMP plans to update the current guidelines on elimination [57], including certification, in 2014. Since the criteria for subnational certification should be consistent with the criteria for WHO national-level certification, WHO-GMP presented only the broad criteria for subnational malaria elimination to MPAC at this September 2013 meeting [58,59]; they will finalize and submit them to MPAC after updating the global guidelines in 2014.

WHO-GMP outlined some of its general principles for subnational elimination: (a) the processes for validation of malaria-free status should emulate the WHO certification scheme; (b) definitions used in WHO elimination and certification guidelines e.g. “malaria free area” are valid for subnational elimination; (c) “certification” based on explicit criteria is preferable to a “declaration”, which may easily become arbitrary; and, (d) elimination, once achieved, should free up resources for areas where malaria is still a public health burden, although there are ongoing resource requirements to prevent reintroduction of malaria.

In addition, the process of achieving subnational elimination should be standardized and officialized; a national team should conduct evaluation and a higher-level experienced commission should be established to evaluate and validate the work of the elimination team. The team should if possible include external international experts so as to enhance the validity and credibility of the process.

The evaluation criteria by which “malaria-free” subnational elimination status would be measured would be: (a) no locally transmitted case in the last three years, at the minimum; (b) a malaria surveillance system set up and implemented with full coverage of the area under consideration; and, (c) a comprehensive plan of action with continued political and financial support to prevent re-establishment of transmission.

The role of WHO in this process would be to provide technical assistance to its Member States, if requested. However, WHO does not have, and is not expected to have, sufficient staff to participate in the certification of all candidate subnational areas in all countries; these would be the sole responsibility of the country itself.

MPAC supported the overall concept of subnational elimination for the many advantages outlined by WHO-GMP. It also voiced strong support for WHO-GMP’s restricted participation in the process, which it saw as a country-led endeavour. Members agreed that the criteria for undertaking subnational elimination should follow the guidelines for national elimination; however they cautioned WHO-GMP over the use of the word “certification”, preferring to restrict it only for national elimination and to use an alternative, such as “validation” or something similar, for subnational malaria-free status confirmation. “Validation” would be a national responsibility that would follow the WHO guidelines for national elimination, but might be modified to country-specific requirements. MPAC concluded that once the guidelines for elimination have been updated, more discussion will be needed on technical issues, such as whether PCR facilities, or an extensive database, will be essential requirements for subnational validation and to what extent the rigorous requirements for national certification of elimination will be adapted for subnational application.

## Discussion

The wording for recommendations were finalized by MPAC during their closed session following the two and a half days of open sessions; conclusions have been included in the summaries of the meeting sessions above, and links to the full set of meeting documents have been provided as references.

Position statements and policy recommendations made by the MPAC are approved by the WHO Director-General, and will be issued formally and disseminated to WHO Member States by WHO-GMP or the WHO Regional Offices. Conclusions and recommendations from MPAC meetings are published in the *Malaria Journal* as part of this series.

MPAC provided suggestions for the agenda for its next meeting to the WHO-GMP Secretariat. In addition to requesting updates from each of its four TEGs (Chemotherapy, Drug Resistance and Containment, Vector Control, and Surveillance, Monitoring and Evaluation), MPAC approved the convening of ERGs on diagnosis in low transmission settings [60] and G6PD testing [61], both of which will report back at its next meeting in March 2014. MPAC also suggested the convening of an ERG on *Plasmodium knowlesi*, which will report back to MPAC at a future meeting.

Feedback from the MPAC meeting will also be given to and received from the global malaria community at the RBM Board meeting in November 2013, through the publication of this article, and subsequent correspondence.

Ongoing engagement with and attendance by interested stakeholders at MPAC meetings continues to be encouraged. In addition to open registration for MPAC meetings, which will continue (via the WHO-GMP website starting in January 2014) and attendance by four standing observers (RBM, the Global Fund, UNICEF, Office of the UN Special Envoy for malaria), the active participation of seven rotating NMCP representatives and all six WHO Regional Malaria Advisors was strongly welcomed.

## Conclusion

The meeting feedback received from participants and observers [62], and MPAC members themselves, was very positive. Having met four times to date, the format of MPAC meetings and its feedback loops with other advisory bodies and stakeholders is fairly settled, although it remains an evolving process. WHO-GMP and the MPAC continue to welcome strongly any feedback, support and suggestions for improvement to MPAC meetings from the global malaria community.

The next meeting of the MPAC will take place from 12 to 14 March, 2014 in Geneva, Switzerland. Further information including the agenda and details on how to register will be made available in January 2014 on the MPAC page of the WHO-GMP website, although questions are welcome at any time [6].

## Endnotes

^a^The complete set of all MPAC September 2013 meeting-related documents including background papers, presentations, and member declarations of interest can be found online at http://www.who.int/malaria/mpac/sep2013/en/index.html.

## Abbreviations

MPAC: Malaria Policy Advisory Committee; WHO-GMP: World Health Organization Global Malaria Programme; NMCP: National malaria control programme; GPIRM: Global Plan for Insecticide Resistance Management; IRS: indoor residual spraying; RDT: Rapid Diagnostic Test; FIND: Foundation for Innovative New Diagnostics; TEG: Technical Expert Group; SMC: Seasonal Malaria Chemoprevention; RBM: Roll Back Malaria; GPARC: Global plan for artemisinin resistance containment; WMR: World Malaria Report; LLIN: Long-lasting insecticide treated nets; ANC: antenatal care; EPI: Expanded Programme on Immunization; MIP: malaria in pregnancy; IPTp-SP: Intermittent Preventive Treatment of malaria in pregnancy using sulphadoxine-pyrimethamine; ERG: Evidence Review Group; MQ: mefloquine; CTX: co-trimoxazole; GTS: Global Technical Strategy 2016–2025; GMAP: Global Malaria Action Plan; ERAR: Emergency Response to Artemisinin Resistance in the Greater Mekong subregion; ACT: Artemisinin-based combination therapy; MAP: Malaria Atlas Project; MIS: Management Information System; PfPR: *P. falciparum* parasite rate; CFR: case fatality rate; SARA: Service Availability and Readiness Assessments.

## Competing interests

The authors declare that they have no competing interests.

## Authors’ contributions

All authors listed under authors informations have equally contributed to the article. All authors have read and approved the final version of the manuscript.

## Authors’ information

WHO Malaria Policy Advisory Committee Members.

• Salim Abdulla, Ifakara Health Institute, Dar Es Salaam, United Republic of Tanzania.

• Pedro Alonso, Centre for International Health and Research, Barcelona, Spain.

• Fred Binka, University of Ghana, Accra, Ghana.

• Patricia Graves, James Cook University, Cairns, Australia.

• Brian Greenwood, London School of Hygiene and Tropical Medicine, London, United Kingdom.

• Rose Leke, University of Yaoundé, Yaoundé, Cameroon.

• Elfatih Malik, Ministry of Health, Gezira, Sudan.

• Kevin Marsh, Kenya Medical Research Institute, Kilifi, Kenya.

• Sylvia Meek, Malaria Consortium, London, United Kingdom.

• Kamini Mendis, Colombo, Sri Lanka.

• Allan Schapira, Legazpi City, Philippines.

• Laurence Slutsker, Centers for Disease Control and Prevention, Atlanta, United States of America.

• Marcel Tanner, Swiss Tropical Public Health Institute, Basel, Switzerland.

• Neena Valecha, National Institute of Malaria Research, New Delhi, India.

• Nicholas White, Mahidol University, Bangkok, Thailand.

WHO Malaria Policy Advisory Committee Secretariat.

• Andrea Bosman, WHO Global Malaria Programme, Geneva, Switzerland.

• Richard Cibulskis, WHO Global Malaria Programme, Geneva, Switzerland.

• Bianca D’Souza, WHO Global Malaria Programme, Geneva, Switzerland and London School of Hygiene and Tropical Medicine, London, United Kingdom.

• Michael Lynch, WHO Global Malaria Programme, Geneva, Switzerland.

• Michael MacDonald, WHO Global Malaria Programme, Geneva, Switzerland.

• Rossitza Mintcheva, WHO Global Malaria Programme, Geneva, Switzerland.

• Abraham Mnzava, WHO Global Malaria Programme, Geneva, Switzerland.

• Robert Newman, WHO Global Malaria Programme, Geneva, Switzerland.

• Pascal Ringwald, WHO Global Malaria Programme, Geneva, Switzerland.

• Zsofia Szilagyi, WHO Global Malaria Programme, Geneva, Switzerland.

• Chansuda Wongsrichanalai, WHO Global Malaria Programme, Geneva, Switzerland.
